# The impact of genetic variants in *IL1R2* on cervical cancer risk among Uygur females from China: A case–control study

**DOI:** 10.1002/mgg3.516

**Published:** 2018-11-20

**Authors:** Fanglin Niu, Tianchang Wang, Jing Li, Mengdan Yan, Dianzhen Li, Bin Li, Tianbo Jin

**Affiliations:** ^1^ Key Laboratory of Resource Biology and Biotechnology in Western China (Northwest University) Ministry of Education Xi’an China; ^2^ Department of Radiotherapy, Shaanxi Provincial Cancer Hospital Affiliated to Medical College Xi’an Jiaotong University Xi’an China; ^3^ Key Laboratory of Molecular Mechanism and Intervention Research for Plateau Diseases of Tibet Autonomous Region, School of Medicine Xizang Minzu University Xianyang China; ^4^ Key Laboratory of High Altitude Environment and Genes Related to Diseases of Tibet Autonomous Region, School of Medicine Xizang Minzu University Xianyang China; ^5^ Key Laboratory for Basic Life Science Research of Tibet Autonomous Region, School of Medicine Xizang Minzu University Xianyang China

**Keywords:** cervical cancer, genetic polymorphisms, *IL1R2*, inflammatory and immune response, population‐based study, Uygur females

## Abstract

**Background:**

Disordered inflammation and immune response is an acknowledged risk factor for cervical cancer development. Interleukin‐1 receptor type 2 (IL1R2) is a decoy receptor for IL‐1 cytokines and involved in host inflammatory and immune progression which could lead to the lesion and neoplasia of cervix. In this study, we aimed to evaluate the relationships between *IL1R2* polymorphisms and cervical cancer risk in Uygur females from China.

**Methods:**

In this case–control study, genotypes of six selected variants (rs11674595, rs4851527, rs719250, rs3218896, rs3218977, and rs2072472) distributed in *IL1R2* were detected among 247 cervical cancer patients and 286 healthy controls with the usage of an Agena MassARRY method. Furthermore, Genetic models and haplotype analyses were conducted to estimate the associations of *IL1R2* polymorphisms with cervical cancer risk.

**Results:**

After statistical analyses, rs719250 (odd ratio [OR] = 1.436, 95% confidence interval [95% CI] = 1.079–1.911, *p* = 0.013) and rs3218896 (OR = 1.552, 95% CI = 1.080–2.229, *p* = 0.017) showed obvious evidence in correlation to cervical cancer susceptibility owing to the surviving significant differences between cases and controls in allele model. Genetic model analyses also revealed significant associations of rs719250 and rs3218896 with cervical cancer risk in the codominant model, the dominant model and the log‐additive model even after adjustment for age (*p* < 0.05). Moreover, haplotype “T/A” of rs11674595/rs4851527 (adjusted OR = 0.73, 95% CI = 0.54–0.98, *p* = 0.037) and “T/C” of rs719250/rs3218896 (adjusted OR = 1.61, 95% CI = 1.10–2.36, *p* = 0.015) exhibited protective and risky effects for Uygur individuals on cervical cancer development, respectively.

**Conclusion:**

Our data first shed the new light on the associations of *IL1R2* polymorphisms with cervical cancer susceptibility among Uygur females. These results are supposed to facilitate the tumorigenesis genetic research among Chinese minorities.

## 1 INTRODUCTION

Cervical cancer, as one of the human malignant diseases, is considered to be a serious threat to females worldwide (Torre et al., 2015). Previous studies have revealed the critical effects of chronic inflammation and immune on cervix tumorigenesis and demonstrated the disorders during these processes (Zidi et al., 2015). While the detailed pathogenic mechanism has not been elucidated completely, multiple cytokines functioning in primary immune and inflammatory response have been confirmed widely involved in the progression from precancerous lesion occurrence to invasive cervical cancer (Chang, Xu, & Fan, 2015; Shi et al., 2014; Zidi et al., 2017). Polymorphisms in these inflammatory‐related genes might result in functional deficiency of their products and eventually facilitate cervical cancer development (Jian, Meng, Shuang, Duan, & Chen, 2016; Lv et al., 2015).

Interleukin‐1 (IL‐1) cluster is tightly related to extensive events in pro‐inflammatory reaction of human through in concert with numerous mediators or cytokines, and serves as a multifunction factor in gynecological cancers (Dinarello, 1996; Zidi et al., 2015). Nevertheless, these inflammatory responses could be controlled by certain corresponding antagonists, which mainly exert anti‐inflammatory impacts and thereby lead to the reduced destruction of cells or tissues and inhibition of immune‐mediated inflammatory diseases, allergies, and even cancer (Palomo, Dietrich, Martin, Palmer, & Gabay, 2015). IL1R2 (Interleukin‐1 receptor type 2, OMIM: 147811) is a decoy receptor which belongs to the interleukin‐1 receptor family. Published research has summarized that IL1R2 can serve as a molecular trap for IL‐1 members and thus inhibit the inflammatory reaction boosted by them (Bessis et al., 2000; Colotta, Dower, Sims, and Mantovani (1994)). IL1R2 acts as an intracellular inhibitor for pro‐IL1‐1α in necrosis‐induced sterile inflammation as well (Zheng, Humphry, Maguire, Bennett, & Clarke, 2013). Although structurally resembling the type 1 IL‐1 receptor (IL1R1), IL1R2 is incapable of signal transduction for the absence of crucial domains (Peters, Joesting, & Freund, 2013). Furthermore, an increasing number of studies provided insights into IL1R2 at genetic level and demonstrated the significant influences of its single nucleotide polymorphisms (SNPs) on occurrence and development of various diseases, including several types of cancer in different populations or genders (Alfaro et al., 2014; Jones et al., 2013; Kamei et al., 2014; Momenzadeh et al., 2016; Na et al., 2017; Stephens et al., 2014). The involvement of IL1R2 has also been reported in the progression of cervical cancer (Niu, Wang, Pei, & Li, 2017). *IL1R2* rs4851527 affects the concentrations of relevant cytokines via interacting with two SNPs in *TLR4*, which contributes to the pro‐inflammatory reaction in a woman's cervix (Ryckman, Williams, Krohn, & Simhan, 2011).

Uygur is a Turkic ethnic group with the majority living in Xinjiang Autonomous Region, whose total population has topped 10 million in China (Jin et al., 2015). Epidemiological investigation has revealed high morbidity and mortality of cervical cancer among Uygur women in recent years and indicated that the occurrence could be attributed to the early childbirth, multiple childbirth, high‐risk sex partner, and suppression of immune function (Ma, Hong, Lu, Chen, & Ma, 2015). Currently, a series of studies have proved the effects of genetic variants within interleukin genes on disease susceptibility in Chinese populations (Jin et al., 2016; Li et al., 2012; Yuan et al., 2012), however, the genetic determinants associated with cervical cancer risk among Chinese Uygur individuals are seldom reported. In this study, we performed a research to identify the risk variations in *IL1R2* with an intention to better explore the characteristic predisposition of cervical cancer in Uygur women.

## 2 MATERIALS AND METHODS

### 2.1 Ethical compliance

Our research has been approved by the ethics committee of People's Hospital of Xinjiang Uygur Autonomous and Northwest University. All procedures performed in this study were in accordance with the 1964 Helsinki declaration and its later amendments. Signed informed consent documents were obtained from all participants included in this study with the ethics committee approval of People's Hospital of Xinjiang Uygur Autonomous and Northwest University.

### 2.2 Subject introduction and sample collection

Without regard to the disease stage restriction, 247 cervical cancer patients at an average age of 54.55 (54.55 ± 10.31 years) were enrolled in this case–control study. All patients were diagnosed with cervical cancer in recent years after a minimum of five small biopsies and endocervical curettage for histopathological and cytomorphological characteristics confirmation. Moreover, blood samples were obtained before the clinical treatment could be conducted. Meanwhile, healthy, unrelated Uygur controls who were ascertained eligible for this exploratory study were randomly recruited from local health checkup center. Detailed clinical data were collected by face‐to‐face questionnaire and clinical record search in order to exclude the individuals with cervical lesions, endometriosis, chronic disease, drug allergy history, gynecological cancer family history and other adverse states on vital systems or organs. Finally, 286 subjects aged about 50.82 (50.82 ± 15.18 years) were selected as control group for further study. All the participants were ethnic Uygur individuals from Xinjiang Province of northwestern China and had at least three generations of exclusive Uygur ancestry. These individuals were considered to be the representative case and control samples of Uygur population according to their characteristic lifestyles and relatively limited places of residence.

### 2.3 DNA isolation and SNP selection

rs11674595, rs4851527, rs719250, rs3218896, rs3218977, and rs2072472 in *IL1R2 *(GenBank reference sequence version number: NC_000002.12) were selected to be demonstrated in this study owing to their minor allele frequencies (MAF) >5% in Uygur women and Hardy–Weinberg equilibrium (HWE) *p* values >0.05 in controls. None of them has been reported previously in correlation to cervical cancer. Genomic DNA was extracted from blood samples with the GoldMag‐Mini Blood Genomic DNA Purification kit (GoldMagLtd., Xi'an, China). DNA concentration was measured at the wavelength of A260 nm by a NanoDrop 2000C spectrophotometer (Thermo Scientific, Waltham, MA, USA). Primers for multiplexed SNP MassEXTEND assay were designed with the application of Agena Bioscience Assay Design Suite software, version 2.0 (https://agenacx.com/online-tools/), and the genotypes of the SNPs were determined following the protocols of MassARRAY Nanodispenser (Agena Bioscience, San Diego, CA, USA) and MassARRAY iPLEX platform (Agena Bioscience) recommended by the manufacturer. Primers used in this study are listed in Supporting Information Table S1. Finally, Agena Bioscience TYPER software, version 4.0 was applied to conduct data management.

### 2.4 Statistical analyses

Preliminary statistical analyses were carried out on SPSS 16.0 (SPSS, Chicago, IL, USA) and Microsoft Excel. To test the departure from HWE for genetic variants in the control group, the observed and expected heterozygosity were compared with Fisher's exact test. Pearson chi‐squared test was performed to evaluate the significant differences on allele and genotype frequencies between cases and controls. The threshold *p* value of statistical significance was considered at 0.05. Using PLINK software, version 1.07 (https://zzz.bwh.harvard.edu/plink/ld.shtml), associations between SNPs and cervical cancer risk were estimated through odd ratios (ORs) and 95% confidence intervals (CIs) under four genetic models: codominant, dominant, recessive, and log‐additive with or without adjustment for age. Additionally, we assessed the pairwise linkage disequilibrium (LD) and risk association of the genetic combinations with Haploview software package, version 4.2 and SHEsis software platform (https://analysis.bio-x.cn/myAnalysis.php).

## 3 RESULTS

This research totally included 533 subjects with Uygur ancestry, which comprised 247 cervical cancer cases and 286 healthy controls. With independent sample *t* test, distribution of age was significantly different between case and control groups (*p* < 0.001, Table 1). Therefore, logistic regression models with adjustment for age were applied to reduce the effect of covariate in this study.

**Table 1:** Distribution of age in cervical cancer cases and healthy controls

	Cases (*n* = 247)	Controls (*n* = 286)	*p*‐value
Age (years)	54.55	50.82	<0.001
Standard deviation	10.31	15.18	

Note

*p*‐value obtained from independent sample *t* test.

Specific information and characteristics of the selected *IL1R2* SNPs (rs11674595, rs4851527, rs719250, rs3218896, rs3218977, and rs2072472) are showed in Table 2. None of them should be removed owing to their conformance with HWE (*p* > 0.05). All SNPs were available for the subsequent association analysis. As depicted in Table 2, the minor allele frequencies (MAF) of the six selected variants ranged from approximately 15.4% to 33.6% and 10.5% to 39.2% in cases and controls, respectively. Notably, the MAF of rs719250 and rs3218896 were observably different in patients when compared with those in healthy group, which indicated the risk roles of these two SNPs in the development of cervical cancer among Uygur females. The minor alleles “T” in rs719250 and “C” in rs3218896 were associated with an increased risk of cervical cancer (rs719250, OR = 1.436, 95% CI = 1.079–1.911, *p = *0.013; rs3218896, OR = 1.552, 95% CI = 1.080–2.229, *p* = 0.017). For age‐adjusted risk models in Table 3, there was no significant difference detected between cases and controls in rs4851527, though a moderate association with a decreased risk of cervical cancer appeared in recessive model (OR = 0.58, 95% CI = 0.34–1.01, *p* = 0.050) and log‐additive model (OR = 0.77, 95% CI = 0.59–1.00, *p* = 0.050) before adjustment*.* It could also be concluded from Table 3 that significantly increased susceptibility to cervical cancer still existed in the age‐adjustment genetic models of *IL1R2* rs719250 (codominant model T/C adjusted OR = 1.97, 95% CI = 1.36–2.85, *p* = 0.001; dominant model adjusted OR = 1.80, 95% CI = 1.27–2.57, *p* = 0.001; log‐additive model adjusted OR = 1.44, 95% CI = 1.07–1.94, *p* = 0.014) and rs3218896 (codominant model T/C adjusted OR = 1.68, 95% CI = 1.12–2.54, *p* = 0.044; dominant model adjusted OR = 1.66, 95% CI = 1.11–2.49, *p* = 0.014; log‐additive model adjusted OR = 1.56, 95% CI = 1.07–2.28, *p* = 0.020). The “TC” genotype of rs719250 was marginally associated with a 1.36‐fold increased cervical cancer risk compared with “CC” genotype. For rs3218896, the same genotype conferred an enhanced cervical cancer risk after considering “TT” genotype as the reference. Dominant model and log‐additive model exhibited undesirable relationships between rs719250, rs3218896 and cervical cancer risk as well. Unfortunately, genetic model analyses of *IL1R2* rs11674595, rs3218977 and rs2072472 did not uncover any significant difference between Uygur patients and healthy controls (*p* > 0.05).

**Table 2:** Basic information and allele frequencies of the selected SNPs in *IL1R2*

SNP	Chromosome	Position	Alleles A<B	Gene	Role	Minor allele frequency		HWE *p* a *‐*value	OR (95% CI)	*p* b‐value
						Case	Control			
rs11674595	chr2	102610992	C<T	IL1R2	Intron	0.211	0.230	1.000	0.899 (0.671–1.204)	0.474
rs4851527	chr2	102622376	A<G	IL1R2	Intron	0.336	0.392	0.536	0.786 (0.612–1.011)	0.060
rs719250	chr2	102623718	T<C	IL1R2	Intron	0.267	0.202	0.139	*1.436 * *(* *1.079* *–* *1.911)*	*0* *.* *013*
rs3218896	chr2	102631652	C<T	IL1R2	Intron	0.154	0.105	1.000	*1.552 * *(* *1.080* *–* *2.229)*	*0* *.* *017*
rs3218977	chr2	102641201	G<A	IL1R2	Intron	0.217	0.224	0.174	0.959 (0.717–1.283)	0.778
rs2072472	chr2	102643019	G<A	IL1R2	Intron	0.227	0.230	0.616	0.983 (0.737–1.309)	0.904

Notes

GenBank reference sequence version number of *IL1R2*: NC_000002.12.

Italics indicates the SNP with statistical significance (*p* < 0.05).

HWE: Hardy–Weinberg equilibrium; IL1R2: interleukin‐1 receptor type 2; OR: odd ratio; SNP: single nucleotide polymorphism; 95% CI: 95% confidence interval.

a *p*‐values obtained from Fisher's exact test.

b *p*‐values obtained from Pearson χ^2^ test.

**Table 3:** Significant genetic variants in *IL1R2* related to the risk of cervical cancer

SNP	Model	Genotype	Control	Case	Crude analysis without adjustment		Adjustment for age	
					OR (95% CI)	*p* a‐value	OR (95% CI)	*p* b‐value
rs4851527 (call rate 100%)	Codominant	G/G	103 (36.0%)	103 (41.7%)	1.00	0.110	1.00	0.210
		G/A	142 (49.7%)	122 (49.4%)	0.86 (0.60–1.24)		0.89 (0.62–1.29)	
		A/A	41 (14.3%)	22 (8.9%)	0.54 (0.30–0.96)		0.59 (0.32–1.06)	
	Dominant	G/G	103 (36.0%)	103 (41.7%)	1.00	0.180	1.00	0.290
		G/A‐A/A	183 (64.0%)	144 (58.3%)	0.79 (0.55–1.12)		0.83 (0.58–1.18)	
	Recessive	G/G‐G/A	245 (85.7%)	225 (91.1%)	1.00	*0* *.* *050*	1.00	0.095
		A/A	41 (14.3%)	22 (8.9%)	*0.58 * *(* *0.34* *–* *1.01)*		0.63 (0.36–1.09)	
	Log‐additive	—	—	—	*0.77 * *(* *0.59* *–* *1.00)*	*0* *.* *050*	0.81 (0.62–1.05)	0.110
rs719250 (call rate 99.62%)	Codominant	C/C	185 (65.1%)	125 (50.6%)	1.00	*6* *.* *00E* *−* *04*	1.00	*0* *.* *001*
		T/C	83 (29.2%)	112 (45.3%)	*2.00 * *(* *1.39* *–* *2.87)*		*1* *.* *97 * *(* *1.36* *–* *2.85)*	
		T/T	16 (5.6%)	10 (4.0%)	0.93 (0.41–2.10)		0.93 (0.41–2.14)	
	Dominant	C/C	185 (65.1%)	125 (50.6%)	1.00	*7* *.* *00E* *−* *04*	1.00	*0* *.* *001*
		T/C‐T/T	99 (34.9%)	122 (49.4%)	*1.82 * *(* *1.29* *–* *2.59)*		*1* *.* *80 * *(* *1.27* *–* *2.57)*	
	Recessive	C/C‐T/C	268 (94.4%)	237 (96.0%)	1.00	0.400	1.00	0.420
		T/T	16 (5.6%)	10 (4.0%)	0.71 (0.31–1.59)		0.72 (0.32–1.62)	
	Log‐additive	—	—	—	*1.46 * *(* *1.09* *–* *1.95)*	*0* *.* *011*	*1* *.* *44 * *(* *1.07* *–* *1.94)*	*0* *.* *014*
rs3218896 (call rate 100%)	Codominant	T/T	229 (80.1%)	174 (70.5%)	1.00	*0* *.* *034*	1.00	*0* *.* *044*
		T/C	54 (18.9%)	70 (28.3%)	*1.71 * *(* *1.14* *–* *2.56)*		*1* *.* *68 * *(* *1.12* *–* *2.54)*	
		C/C	3 (1.0%)	3 (1.2%)	1.32 (0.26–6.60)		1.21 (0.23–6.25)	
	Dominant	T/T	229 (80.1%)	174 (70.5%)	1.00	*0* *.* *010*	1.00	*0* *.* *014*
		T/C‐C/C	57 (19.9%)	73 (29.6%)	*1.69 * *(* *1.13* *–* *2.51)*		*1* *.* *66 * *(* *1.11* *–* *2.49)*	
	Recessive	T/T‐T/C	283 (99.0%)	244 (98.8%)	1.00	0.860	1.00	0.940
		C/C	3 (1.0%)	3 (1.2%)	1.16 (0.23–5.80)		1.07 (0.21–5.50)	
	Log‐additive	—	—	—	*1.59 * *(* *1.09* *–* *2.31)*	*0* *.* *014*	*1* *.* *56 * *(* *1.07* *–* *2.28)*	*0* *.* *020*

Notes

Italics indicates the SNP with statistical significance (*p* < 0.05).

IL1R2: interleukin‐1 receptor type 2; OR: odds ratio; SNP: single nucleotide polymorphism; 95% CI: 95% confidence interval.

a *p*‐values calculated by logistic regression analysis.

b *p*‐values calculated by logistic regression analysis with adjustment for age.

**Figure 1:**
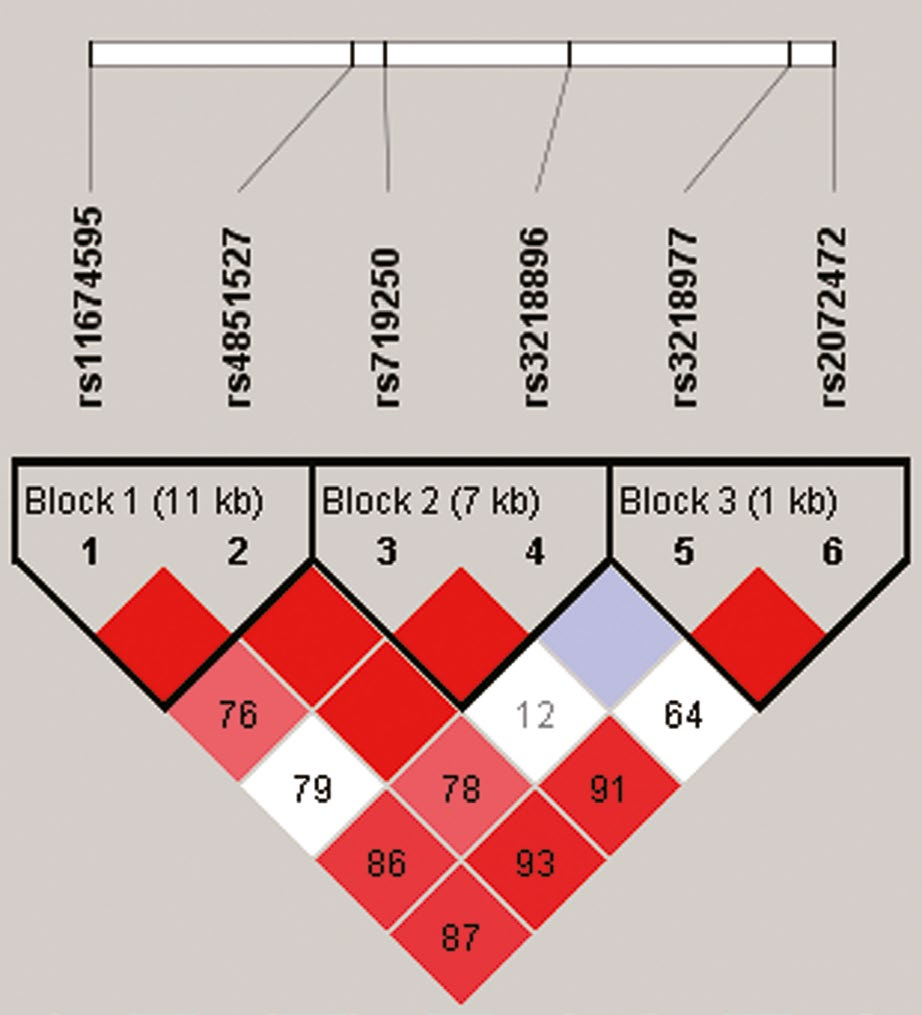
Linkage disequilibrium (LD) analysis of the six single nucleotide polymorphisms (SNPs) in interleukin‐1 receptor type 2 (*IL1R2*). Three haplotype blocks are formed by rs11674595 and rs4851527 (Block 1, 11 kb), rs719250 and rs3218896 (Block 2, 7 kb), rs3218977 and rs2072472 (Block 3, 1 kb), respectively. Standard color schemes indicate different levels of LD. Bright red: LOD > 2, D′ = 1; Pink red: LOD < 2, D′ < 1; Blue: LOD < 2, D′ = 1; White: LOD < 2, D′ < 1; LOD: Log of the odds of there being LD between two loci.

Furthermore, the association between cervical cancer risk and haplotype of *IL1R2* was analyzed in this work. In Figure 1, three LD blocks in *IL1R2* formed by rs11674595‐rs4851527, rs719250‐rs3218896, and rs321897‐rs2072472 were ascertained at 11, 7, and 1 kb, respectively. For haplotype analysis displayed in Table 4, rs11674595/rs4851527 “T/A” (adjusted OR = 0.73, 95% CI = 0.54–0.98, *p* = 0.037) was deemed to be a favorable factor owing to the lower susceptibility of carriers to cervical cancer while the haplotype rs719250/rs3218896 “T/C” (adjusted OR = 1.61, 95% CI =1.10–2.36, *p* = 0.015) was exactly opposite. However, there were no statistical differences in haplotypes of rs3218977/rs2072472, which was indicative of non‐correlation to the risk of cervical cancer.

**Table 4:** Haplotype analysis of the variants in *IL1R2* and the association with cervical cancer risk

	Haplotype		Frequency	Crude analysis without adjustment		Adjustment for age	
				OR (95% CI)	*p* a‐value	OR (95% CI)	*p* b‐value
Block 1	rs11674595	rs4851527					
	T	G	0.4136	1.00	—	1.00	—
	T	A	0.3659	*0.70 * *(* *0.52* *–* *0.93)*	*0* *.* *015*	*0.73 * *(* *0.54* *–* *0.98)*	*0* *.* *037*
	C	G	0.2205	0.76 (0.55–1.05)	0.100	0.77 (0.56–1.07)	0.120
Block 2	rs719250	rs3218896					
	C	T	0.7679	1.00	—	1.00	—
	T	C	0.1276	*1.64 * *(* *1.12* *–* *2.39)*	*0* *.* *010*	*1.61 * *(* *1.10* *–* *2.36)*	*0* *.* *015*
	T	T	0.1045	1.28 (0.86–1.91)	0.220	1.28 (0.86–1.92)	0.230
Block 3	rs3218977	rs2072472					
	A	A	0.5510	1.00	—	1.00	—
	A	G	0.2285	0.96 (0.71–1.31)	0.810	0.94 (0.69–1.29)	0.700
	G	A	0.2205	0.94 (0.69–1.30)	0.720	0.89 (0.64–1.23)	0.480

Notes

Italics indicates the haplotype with statistical significance (*p* < 0.05).

IL1R2: interleukin‐1 receptor type 2; OR: odds ratio; 95% CI: 95% confidence interval.

a *p*‐values calculated by logistic regression analysis.

b *p*‐values calculated by logistic regression analysis with adjustment for age.

## 4 DISCUSSION

Inflammation and immune response has been demonstrated in relation to the tumorigenesis and metastasis through a complicated dynamic system consisting of various cytokines, cytokine receptors, and downstream targets (Jones et al., 2013). The interleukin‐1 cytokine family, members of which engage in innate immune and inflammatory processes, comprises both pro‐inflammatory agonists and defined or potential antagonists. Previous reports have discussed the inhibitory regulation of IL‐1 cytokines activities and emphasized that clinical syndromes induced by host auto‐inflammatory disorders should be ascribed to an imbalance of agonist‐antagonist resulting from genetic variations (Palomo et al., 2015). Genetically, it has been well‐studied in numerous diseases that IL1R2 mediating diverse inflammatory and immune responses as an IL‐1 inhibitor, however, the influence of *IL1R2* polymorphic SNPs was seldom reported on minorities in China. In our research, a case–control study was carried out to evaluate the effects of polymorphisms in *IL1R2* on cervical cancer susceptibility among Uygur women from northwestern China for the first time. Considerable associations were detected between *IL1R2* rs719250, rs3218896 and cervical cancer among Uygur females. Linkage disequilibrium and haplotype analysis also provided evidence for the roles of different haplotypes formed by variants in cervical cancer susceptibility determine. These results yield a new insight of *IL1R2* in cervical cancer development in Uygur females.

The type 2 IL‐1 receptor (IL1R2) plays a pivotal role in mediating the biological activities of IL‐1 cytokine with both membrane‐bound and soluble forms (Smith et al., 2003). When functioning in IL‐1 signaling interference, IL1R2 could be enhanced in expression level by several immunosuppressive and anti‐inflammatory medicaments (Peters et al., 2013). A recent publication has revealed that *IL1R2* expression is increased in human CD4+ T‐cells with age (Taylor et al., 2017) and up‐regulated during T‐cell activation process of CD3+, CD4+, and CD8+ in immune responses (Dirk, Min, & Papoutsakis, 2008). Basing on the determination for density changes of immune cells in cervical cancer, researchers have concluded T‐cells’ involvement in cervical pre‐neoplastic lesions development and cervical cancer progression, especially for CD8+, whose count significantly increased in stroma (Bedoya et al., 2013). These facts suggest distinct relationships among IL1R2, T‐cell activity, and cervical cancer. Furthermore, dysregulation of IL1R2 has been detected in high‐grade squamous intraepithelial lesion (HSIL) and squamous cell carcinomas (SCC) in invasive cervical cancer, which ascertains its implication in cervical tumor progression (Niu et al., 2017).

There is increasing interest in the possibility that polymorphisms are responsible for a significant proportion of heritable human phenotypic variation, including human disease. *IL1R2* polymorphisms have been considered as genetic factors associated with cervical cytokine concentrations in African American and European American women, and the changed cytokine level was related to vaginal disorder. Published report has revealed a distinct role of rs4851527 in *IL1R2* associated with pro‐inflammatory cytokine concentrations at cervix through interacting with SNPs in *TLR4* (rs1554973 or rs7856729) (Ryckman et al., 2011). According to our findings, the “A” allele in rs4851527 and “T” allele in rs11674595 constituted a significant haplotype which leaded to a decreased risk of cervical cancer. In contrast, haplotype “TC” of rs719250 and rs3218896 alerted us a rising risk among Uygur women. Interestingly, rs719250 and rs3218896 also showed negative associations with cervical cancer risk, respectively, under codominant, dominant, and log‐additive genetic models after adjusting for age, which implied the undesirable effects of potential genetic factors in IL1R2 on cervical cancer development. According to the fact that all of the genetic variants are located in the intron region of *IL1R2*, we speculated that these allelic variations might alter the splicing form or transcription level of mRNA and thereby exert influences on the quantity and quality of gene expression, which eventually determines the individual susceptibility to cervical cancer among Uygur women. These findings provided us new insights into the relationship between *IL1R2* polymorphisms and tumorigenesis.

Several potential limitations in the current research should be acknowledged. First, the sample size in this study might be small and a larger sample size is worthy of consideration to validate our results. Second, statistically analyses on other variables such as body mass, dietary preference, and HPV infection were not performed owing to the lack of relevant data. Third, selection bias still existed during the investigation. Accordingly, further studies with larger sample and specific information of potential confounders are warrant to confirm these preliminary findings.

Despite the limitations, this is the first work that concentrates on the polymorphisms of *IL1R2* in correlation to cervical cancer risk in Uygur women from Northwest China. Our results suggested the involvement of *IL1R2* polymorphisms in the development of cervical cancer and provided new genetic markers for cervical cancer susceptibility assessment in the future.

## 5 CONCLUSIONS

In this study, four SNPs in *IL1R2* were demonstrated to be associated with cervical cancer in Uygur females, with protective or risky effects according to the genetic model and haplotype analysis. This study could contribute to the cervical tumorigenesis research for Uygur females and the identification of innate genetic factors for cancer development in Chinese minorities.

## CONFLICT OF INTEREST

None declared.

## AUTHOR CONTRIBUTION

Fanglin Niu, Tianchang Wang, Jing Li, Mengdang Yan, Dianzhen Li, Bin Li, and Tianbo Jin conceived and designed this research. Fanglin Niu, Dianzhen Li, and Bin Li collected the blood samples and all members performed the experiments. Mengdan Yan, Tianchang Wang, and Jing Li analyzed the data. Fanglin Niu, Tianchang Wang, Jing Li, Mengdan Yan, and Tianbo Jin discussed the results and wrote the manuscript. All authors contributed critically to the drafts and approved the final manuscript.
