# Supplementary material for: The impact of genetic variants in IL1R2 on cervical cancer risk among Uygur females from China: A case–control study
Source: Mol Genet Genomic Med. 2018 Nov 20;7(1):e00516. doi: 10.1002/mgg3.516 (PMC6382450; doi:10.1002/mgg3.516)
Supplement: Supplementary file 1 [file MGG3-7-na-s001.docx]

**Supplementary Table S1** Primers used for identification of the *IL1R2* polymorphisms

| SNP | First PCRP (5'-3') | Second PCRP (5'-3') | UEP (5'-3') |
| --- | --- | --- | --- |
| rs11674595 | ACGTTGGATGGAATCACTGGTGGGCTTATG | ACGTTGGATGAATGCAGATTCTCAGGTCGC | GGGACCAACCAGGACTTACTGAATC |
| rs4851527 | ACGTTGGATGAAGGGCTTTGGAATCACCAG | ACGTTGGATGTGGCCGAGATCTTACAGCTA | CTTACAGCTAGTAAGCAGA |
| rs719250 | ACGTTGGATGATCTGACACTCCAGTCTTTG | ACGTTGGATGATCCCAGGGAGAAAAGCAAC | GAGCTTGTACAAGTTTATGAA |
| rs3218896 | ACGTTGGATGCTGCATGTGGATATGGTTTC | ACGTTGGATGCAAAAGGGCTTATGCCTTCC | CCCGCATACTCCAACTTC |
| rs3218977 | ACGTTGGATGTGAGAACTCTGTGGGTTTCG | ACGTTGGATGACATCCAACAGTTTGGAATC | TGTGGTATGTGGGTCA |
| rs2072472 | ACGTTGGATGCTTCGAAATACTCTGTCTGC | ACGTTGGATGTTCTAGAGGCCATGCGAAGA | GCTGCCTTGGGTCACT |

**SNP: single nucleotide polymorphism; PCRP: PCR primer; UEP: Unextended mini sequencing primer.**
